# An Embodied Multi-Sensor Fusion Approach to Visual Motion Estimation Using Unsupervised Deep Networks

**DOI:** 10.3390/s18051427

**Published:** 2018-05-04

**Authors:** E. Jared Shamwell, William D. Nothwang, Donald Perlis

**Affiliations:** 1 Sensors and Electron Devices Directorate, US Army Research Laboratory, 2800 Powder Mill Rd, Adelphi MD 20783, USA; william.d.nothwang.civ@mail.mil; 2 Department of Computer Science, University of Maryland, A.V. Williams Building, College Park, MD 20740, USA; perlis@cs.umd.edu

**Keywords:** deep learning, sensor fusion, optical flow

## Abstract

Aimed at improving size, weight, and power (SWaP)-constrained robotic vision-aided state estimation, we describe our unsupervised, deep convolutional-deconvolutional sensor fusion network, Multi-Hypothesis DeepEfference (MHDE). MHDE learns to intelligently combine noisy heterogeneous sensor data to predict several probable hypotheses for the dense, pixel-level correspondence between a source image and an unseen target image. We show how our multi-hypothesis formulation provides increased robustness against dynamic, heteroscedastic sensor and motion noise by computing hypothesis image mappings and predictions at 76–357 Hz depending on the number of hypotheses being generated. MHDE fuses noisy, heterogeneous sensory inputs using two parallel, inter-connected architectural pathways and *n* (1–20 in this work) multi-hypothesis generating sub-pathways to produce *n* global correspondence estimates between a source and a target image. We evaluated MHDE on the KITTI Odometry dataset and benchmarked it against the vision-only DeepMatching and Deformable Spatial Pyramids algorithms and were able to demonstrate a significant runtime decrease and a performance increase compared to the next-best performing method.

## 1. Introduction

Due to both the speed and quality of their sensors and restrictive on-board computational capabilities, current state-of-the-art (SOA) size, weight, and power (SWaP) constrained autonomous robotic systems must trade operational speed for safety and robustness. This trade-off is especially pronounced in dynamic, GPS-/communications-denied environments where robust navigation must be performed only with on-board sensors and computational resources.

GPS-denied navigation for SWaP constrained unmanned aerial vehicles (UAVs) is commonly performed with a dead-reckoning technique called visual odometry (VO). In VO, motion of the UAV is inferred by tracking the visual motion of objects in the visual scene on a camera’s imaging plane. With a sufficient number of tracking points, an estimate of camera motion (and by extension UAV motion) can be computed [1]. Thus, for VO, a crucial early-step is finding a correspondence mapping between scene elements perceived on the imaging plane in sequentially captured image frames. In the literature, this is referred to as the correspondence problem (see [2] for a review).

Here, we show how additional contextual information embedded in the form of early sensor fusion can greatly reduce the computational burden of the first step in VO: the estimation of pixel-level correspondences between images. We argue that traditional, bottom-up, vision-only approaches aimed at resolving the correspondence problem neglect other available sources of complementary information and show how even noisy extramodal estimates of motion can be leveraged for visual correspondence estimation with reduced runtime.

We have previously shown that our DeepEfference (DE) [3] architecture can efficiently fuse visual information with motion-related information to greatly decrease runtime (20,000%) with minimal performance degradation (12%) for dense image correspondence matching. However, in our previous work, we used motion estimates as inputs to DE that were accurate to within approximately 10 cm of actual pose. In the real-world, systems will rarely have access to comparatively clean signals. Additionally, real noise sources are often heteroscedastic and input-dependent. With the original DEs fast runtime, we saw the possibility of generating many different hypothetical outputs for each input image and then selecting the most accurate at execution time. By learning how to produce *n* image reconstruction predictions, we have extended DE into the Multi-Hypothesis DeepEfference (MHDE) [4] architecture to better handle real-world noise sources.

In this work, we describe new experiments with our MHDE architecture and demonstrate how a multi-hypothesis deep neural network approach can mitigate error introduced from noisy sensor measurements by implicitly learning a non-parameterized noise distribution from which the network then also learns how best to sample. Measurement error, as would be found in real-world applications, can thus be estimated by the network and the highest accuracy network output can then be chosen at inference time. It should be emphasized that the aim of the network is not to explicitly estimate the error on noisy motion estimates, but rather to learn how best to combat this error by generating possible outputs that could best represent the true, uncorrupted motion estimate.

We have expanded upon our previous approaches and now for the first time demonstrate superior performance compared to existing SOA deep dense correspondence approaches in the noise-free case and similar performance in low-noise conditions while maintaining the fast runtime that was a hallmark of our previous approaches.

The main contributions of this paper and the models here described are:

1. Increased mean predictive performance of 16.8% of pixel-position root mean squared error (RMSE) compared to DeepMatching (DM) [5] with a runtime decrease of 3105% (6512% with pruning);
2. Increased mean predictive performance of 17.4% of pixel-position RMSE compared to our previous approach [4] with identical runtime after pruning (158 Hz); and
3. New results and analysis from networks trained with up to 20 hypothesis sub-pathways.

The remainder of the paper is organized as follows: Section 2 describes the motivations for MHDE; Section 3 presents background and related work; Section 4 outlines our deep network approach to fusing noisy heterogeneous sensory inputs and describes the MHDE architecture; Section 5 outlines our experimental and evaluation approaches; Section 6 presents and discusses our experimental results; and Section 7 offers a summary and concluding thoughts.

## 2. Motivations

The sensing and processing pipelines of autonomous and semi-autonomous robotic systems pose a fundamental limit on how fast they may safely travel through an environment. For example, when moving at 20 m/s, a 30 Hz sensor-derived state estimate update rate means that a given robot will travel 0.66 m between state updates. While traveling those 0.66 m, the robot will effectively be blind to any unexpected changes in the environment (e.g., a tree branch blown by a wind gust or an unexpectedly opened door). As a result, current SWaP constrained autonomous and semi-autonomous robotic systems are forced to move very slowly through dynamic, unstructured environments.

SWaP constrained VO pipelines are forced to use lightweight and sparse feature matching approaches for determining visual correspondence that are out-performed by computationally heavier SOA approaches. For example, the visual matching algorithm DM has enabled SOA matching and optical flow [6], but the correspondence-finding step alone can require from 16 s to 6.3 min per RGB image pair depending on the parameter regime used for matching [5]. For real-time operation on SWaP constrained systems, correspondence must be computed orders of magnitude faster (e.g., a minimum of 33 ms per matching pair for a 30 frames per second (FPS) camera commonly used for SWaP constrained robotic applications).

The slow operational speeds of SWaP constrained autonomous systems are especially pronounced for mobile robots operating in dynamic, GPS-/communications-denied environments where safe navigation must be performed only with on-board sensors and computational resources. As mentioned earlier, additional context can aid in reducing the computational burden of real-time state estimation and enable both higher-quality and lower-latency estimates. One way to provide context is by fusing measurements from multiple sensory modalities. However, intelligently integrating multimodal information into low-level sensory processing pipelines remains challenging, especially in the case of SWaP constrained robotic systems.

## 3. Related Work

### 3.1. Visual Odometry and Multi-Sensor Fusion

A known failure mode for VO is in highly dynamic scenes. VO algorithms are subject to the static scene assumption, whereby additional error is introduced when independently moving points in the scene move inconsistently with their dependently moving neighbors. Feedback outlier detection approaches based on algorithms such as random sample consensus (RANSAC) [7] seek to discover the most likely motion that has caused a given transform. However an unconstrained key-point match between two images across a large temporal window and spatial extent is at least exponentially complex [8]. By fusing sensor information from separate modalities, we can effectively constrain the matching process.

Constraining the matching process to be consistent with a narrow range of transforms gleamed from another modality can lead to increased VO performance relative to computational requirements and processing time. Previous work has applied extra-visual feedback signals from inertial measurement units (IMUs) or GPS [9,10] to constrain the matching process. Simple motion models [11,12] have also been used to predict future images based on previously observed image motion. These approaches have been extended to use quadratic motion models [13], which showed improved performance in specific environments (e.g., on flat roads). However, these models implicitly sacrifice responsiveness as they wait for changes in an underlying sensory distribution rather than detecting dominant motion from a separate extra-visual modality.

### 3.2. Deep Spatial Transformations

As mentioned earlier, the correspondence problem has traditionally been tackled with closed-form, analytical approaches (see [2] for a review), but, recently, deep, bio-inspired, solutions have also begun to show promise. These deep approaches solve the correspondence problem by learning to estimate the 3D spatial transformations between image pairs.

In VO as well as many other vision tasks such as motion understanding and stereopsis, a key challenge is discovering quantitative relationships between temporally or spatially adjacent images. Within the last decade, bio-plausible approaches for the visual task of object recognition have set new benchmarks and are now the defacto standard. We agree strongly with Memisevic that bio-plausible, local filtering-based approaches similarly hold promise for the correspondence problem [14].

In computer vision, Siamese-like deep network architectures such as those based on multiplicative interactions have been used successfully for relationship learning between images [14,15,16,17,18,19]. However, there are two problems with these and other deep approaches (e.g., the DeepMatching [5,6] algorithm described earlier) to image transformation learning.

First, these approaches require expensive computation on both initial and target images. They employ Siamese architectures that require parameter-heavy learning and expensive computations to be performed on both source and target images. For SWaP-constrained robots, the number of computational operations required by these Siamese networks must be significantly reduced. Approaches such as L1 and group lasso-based pruning [20,21,22] offer potential mechanisms to reduce the size of networks but fundamentally still require extensive computation on both source and target images.

Second, these approaches do not provide a mechanism to include extra information from another modality as a motion prior while maintaining end-to-end trainability. For robotic applications, heterogeneous sensor information is often available that can be leveraged and may allow for reduced computational constraints and increased performance (see Section 3.3).

### 3.3. Extra-Modal Motion Estimates and Heteroscedastic Noise

Unlike algorithms in pure computer vision domains, algorithms intended for robotic applications need not rely solely on vision. For example, when estimating a robotic system’s egomotion by tracking changes in feature point locations on a robot’s camera’s imaging plane, additional non-visual motion estimates can be fused with visual information (i.e., to bias or serve as a motion prior) to improve egomotion estimation. In real-world systems, additional non-visual motion estimates can be derived from measurements taken from IMUs, GPS, LIDARs, ultrasonic ranging sensors, or the actual input motor commands given to the system.

## 4. Approach

MHDE is an unsupervised deep heterogeneous neural network that employs multiple separable pathways to fuse noisy, heterogeneous sensory information and predict how source images correspond to unseen target images. MHDE effectively reverses the prediction pipeline—rather than using the previous image to reconstruct the future image, it uses the target image to reconstruct the source image. The network receives a noisy estimate of the change in a 3D camera position between source and target frame acquisitions and learns:

1. 2D affine transformation parameters that are applied as a global spatial transform; and
2. local, pixel-level shifts that encapsulate aberrations due to varied scene depth, non-rigid scene objects, etc.

The affine transformations and localized shifts are learned and applied via two interconnected architectural pathways: one for determining global 2 × 3 affine 2D transformation matrices, and a second encoder–decoder pathway that predicts localized, pixel-level shifts that are not captured by the global, approximated 2D affine transformation (see [3] for more information on the DE architecture).

**Figure 1:**
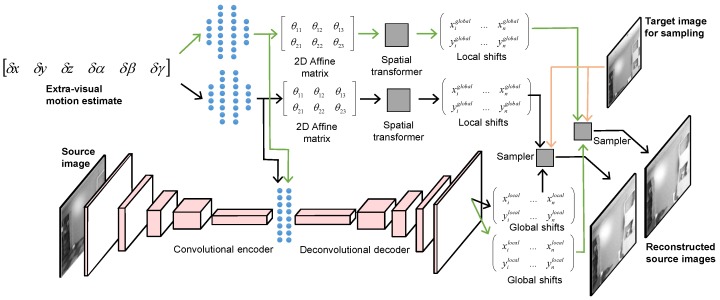
MHDE network diagram depicting an architecture that computes two hypotheses. We experimented with architectures that generated up to 20 hypotheses.

Figure 1 depicts the MHDE network architecture. At the top left, the network receives an extra-visual motion estimate, which is used to estimate the 2D affine transformations mentioned above through its global pathway. MHDE’s local pathway is depicted in the bottom-half of Figure 1. This pathway receives a source image as input and estimates localized, pixel-level shifts that cannot be explained by a global 2D affine transformation (e.g., parallax). Additional details on these two pathways are provided below in Section 4.2 and Section 4.3.

Unlike the original DE, MHDE generates several hypothetical reconstructions that enable increased robustness to noisy inputs. Thus, while DE only has two architectural pathways, MHDE has the same two architectural pathways plus *n* additional hypothesis generation sub-pathways (2–20 in this work) that operate on the outputs of the global and local pathways. This can be seen in Figure 1 where two hypothesis reconstructions are shown.

### 4.1. Winner-Take-All (WTA) Loss Rule

MHDE generates multiple hypothesis reconstructions to enable robustness to stochastic, heteroscedastic, input noise such as found in the real world. The previous DE architecture that generated only a single predicted reconstruction used Euclidean error to train the network by minimizing the loss function

(1) $$L\left(\theta ,I_{t},I_{s}\right)=\underset{\theta }{\mathrm{argmin}}{∥I_{r}\left(\theta ,I_{t}\right)-I_{s}∥}^{2},$$

where $I_{r}$ is an image reconstruction, $I_{t}$ is the image target, and $I_{s}$ is the image source being reconstructed.

If, instead of generating a single reconstruction $I_{r}$, the network generated *n* reconstructions $I_{r}^{i},i\in N$, the loss rule would need to be expanded to train across all hypothesis pathways in the new network. A naive way to compute error for such a multi-hypothesis network would be to simply sum the Euclidean error from all hypotheses and divide by the total number of hypotheses. Then, the network would be trained by minimizing the loss function

(2) $$L\left(\theta ,I_{t},I_{s}\right)=\underset{\theta }{\mathrm{argmin}}\frac{\sum_{i}^{N}{∥I_{r}^{i}\left(\theta ,I_{t}\right)-I_{s}∥}^{2}}{N},$$

where $I_{r}^{i}$ is a hypothesis image reconstruction and the remaining terms are the same as before.

The naive multi-hypothesis loss rule of Equation (2) would lead the network to optimize all pathways simultaneously with each update. However, this may not be optimal for increased robustness to noise. Effectively, we desire the network to generate distinct predictive hypotheses by sampling from a noise distribution that the network implicitly learns. For example, consider when the network has perfectly optimized the loss function of Equation (2):

(3) $$L\left(\theta ,I_{t},I_{s}\right)=\frac{\sum_{i}^{N}{∥I_{r}^{i}\left(\theta ,I_{t}\right)-I_{s}∥}^{2}}{N}\approx 0.$$

In this case, ${∥I_{r}^{i}\left(\theta ,I_{t}\right)-I_{s}∥}^{2}\approx 0,\forall i\in N,$ which means that each hypothesis reconstruction $I_{r}^{i}\left(\theta ,I_{t}\right)$ is approximately equal. As the network is trained and converges to a local minima, loss will affect parameters in each pathway approximately equally and drive outputs from all pathways to a common approximate solution. This is the opposite of what we want from MHDE. Effectively, such a loss rule is equivalent to the standard Euclidean loss rule used in [3] where a single prediction is generated and fails to leverage the multiple outputs that can be generated by MHDE.

To leverage its multiple outputs, we train MHDE using what we call a winner-take-all (WTA) Euclidean loss rule:

(4) $$I_{r}^{*}\left(\theta ,I_{t}\right)⟵\underset{i}{\mathrm{argmin}}{∥I_{r}^{i}\left(\theta ,I_{t}\right)-I_{s}∥}^{2},$$

(5) $$L\left(\theta ,I_{t},I_{s}\right)={∥I_{r}^{*}\left(\theta ,I_{t}\right)-I_{s}∥}^{2},$$

where $I_{r}^{*}$ is the lowest error hypothesis. Loss is then only computed for this one hypothesis and error is backpropagated only to parameters in that one pathway. Now, only parameters that contributed to the winning hypothesis are updated and the remaining parameters are left untouched.

During inference, MHDE selects the hypothesis with the lowest mean cumulative photometric error (Equation (5)) as the relevant hypothesis at inference time. In other words, MHDE selects the hypothesis reconstruction image with the lowest error between its own pixel intensities and those of the original source image.

### 4.2. Pathway 1: Global Spatial Transformer

Spatial transformer (ST) modules [23] apply parametrized geometric transformations to feature-maps (either data inputs or intermediate outputs) in deep networks. The parameters for these transformations (2D affine transformations in our case) can be directly provided to the network as input or can be learned and optimized alongside the other network parameters (e.g., network weights and biases).

MHDE was provided with estimates of the true 3D transformation between source and target images (δx, δy, δz, δα, δβ, δγ). Note, however, that the visual input to MHDE was a single grayscale source image without any depth information. Even if the provided 3D transformation was noise-free and perfectly accurate, it is not possible to analytically perform a 3D warp (assuming translation) on a 2D image due to unknown scene depth at each pixel location. Thus, MHDE approximated 3D warps as 2D affine transformations through a linear-nonlinear optimization using four fully-connected layers, each followed by an additional rectified linear unit (ReLU) [24] nonlinearity layer.

We modified the standard ST module in tensorflow [25] by splitting the layer into two layers—one to perform the affine transformation on grids of source pixel locations $\left(x^{s},y^{s}\right)$ and output target pixel coordinates $\left(x^{t},y^{t}\right)$ and a second layer to perform bilinear sampling given pixel coordinates and an image to sample from.

Although the sampling component of our ST module takes an input image as input, no learn-able parameters are based on input image content and thus our global pathway is a function only of the input transformation estimate and is image content-independent.

### 4.3. Pathway 2: Local, Pixel-Level Shifter

The pixel-level encoder/decoder pathway refines the ST estimate from the first pathway and provides localized estimates of pixel movement to account for depth, non-rigidity, etc.

We implemented this pathway as a convolutional-deconvolutional encoder–decoder. First, the convolutional encoder compresses a source image through a cascade of convolutional filtering operations. The output of the convolutional encoder is concatenated with intermediate outputs from the fully-connected layers from the first, global pathway (the black and blue vertical lines in the center of Figure 1). This concatenated representation is then expanded using a deconvolutional decoder to generate *n* pairs of $\left(x^{t^{\prime }},y^{t^{\prime }}\right)$ pixel locations that are summed with the target pixel coordinates $\left(x^{t},y^{t}\right)$ from the global pathway before bilinear sampling (see [3] for more details).

## 5. Experimental Methods

We conducted experiments with MHDE using five different noise conditions and five different architectures. All architectures were based on DE [3] and implemented both global pathway and local pathways. The main difference between architectures was the number of hypotheses that each was allowed to learn. MHDE was evaluated on the KITTI Odometry dataset [26] and results were benchmarked against correspondence matching results from the SOA DM [5] and Deformable Spatial Pyramids (DSP) approaches [27].

We experimented with four noise conditions where α was 0.0 , 0.1, 0.25, 0.5, or 1.0. We trained networks with 1, 2, 4, 8, 12, 16, 18, or 20 hypothesis generation pathways. For each noise and and hypothesis combination, we trained three networks for a total of 105 different networks.

### 5.1. Noise

Motion estimates exhibit heteroscedastic noise properties where larger movements generate larger amounts of noise [28]. Any approach that seeks to leverage extra-modal motion estimates needs to be robust to real-world heteroscedastic noise.

**Figure 2:**
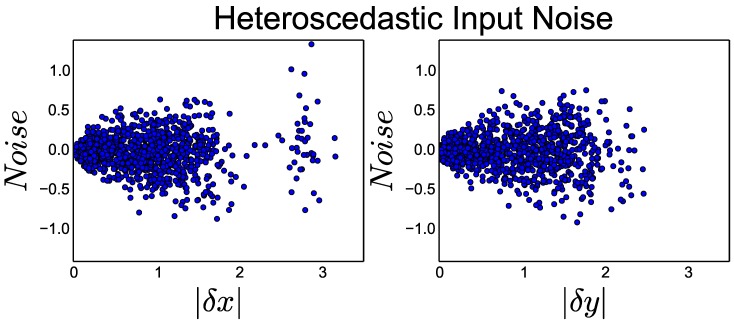
Heteroscedastic noise as a function of transform magnitude for the *X* and *Y* components of the transform input over the test set for a network with a noise parameter α=0.25.

As shown in Figure 2, we simulated real-world noise conditions by applying heteroscedastic noise to each transform input. For each transform T=(δx,δy,δz,δα,δβ,δγ), we introduced heteroscedastic noise to create network input $T^{*}$ according to:

(6) $$T^{*}=T+N\left(0,\alpha \sqrt{T}\right),$$

where α was a constant modifier that was either 0.0, 0.1, 0.25, 0.5, or 1.0.

### 5.2. Evaluation

As in [3], we evaluated MHDE on the KITTI Visual Odometry dataset [26]. KITTI is a benchmark dataset for the evaluation of visual odometry and LIDAR-based navigation algorithms. Images in KITTI were captured at 10 Hz from a Volkswagen Passat B6 as it traversed city, residential, road, and campus environments in Karlsruhe, Germany. Groundtruth poses at each camera exposure were provided by an Real Time Kinematic (RTK) GPS solution and depth is provided with coincident data from a Velodyne laser scanner (Adelphi, MD, USA).

We evaluated MHDE by measuring pixel-level RMSE of MHDE projections of keypoints from source images to target images. The projection errors for each method compared to groundtruth projections were used to determine the mean pixel-level RMSE for each method. For our experiments, ground truth is the new pixel coordinate of a given point in the scene following camera motion.

To calculate these new coordinates, we need (1) the depth of each point in the scene; (2) the 3D transformation between camera poses; and (3) the camera matrix and and transforms between each frame.

In KITTI, all objects in the visual scenes are rigid, thus fulfilling the static scene assumption and allowing for ground truth to be computed from scene depth and camera position. With access to scene depth and true camera pose for KITTI, groundtruth pixel shifts were calculated by applying a 3D warp to 3D pixel locations in the source images to generate the expected pixel locations in the target images. We projected each 3D point in the frame of $camera_{t0}$ to the world frame using the derived projection matrix for $camera_{t0}$ and then reprojected these points in the world frame to $camera_{t1}$ using the inverse projection matrix for $camera_{t1}$. Finally, we transformed points in the frame of $camera_{t1}$ to the image plane. This resulted in a correspondence map between pixel locations in $camera_{t0}$ and $camera_{t1}$ for each point where depth was available (e.g., when depth was outside of the Velodyne laser scanner’s range).

#### Performance Comparisons

We evaluated MHDE pixel-correspondence predictions on the KITTI Visual Odometry dataset [26] against correspondences computed from:

1. Ground truth;
2. The DeepMatching algorithm [5]; and
3. The Deformable Spatial Pyramid Matching algorithm [27].

### 5.3. Training

For training and evaluation, data was separated into train (80%) and test (20%) sets. We used a total of 23,190 image pairs with 80%(18,552) for training and 20%(4638) for testing. In all experiments, we randomly selected an image for the source, used the successive image for the target, and subtracted the two 6-degrees of freedom (DOF) camera poses for the transform input. For each image in each dataset, we cropped the middle 224 × 224 pixel region for network inputs.

We trained MHDE for 200,000 iterations on KITTI Odometry scenes 1–11 for all experiments. We used the Adam solver with batch size = 32, momentum1 = 0.9, momentum2 = 0.99, gamma = 0.5, learning rate = 1 × 10${}^{-4}$, and an exponential learning rate policy for all experiments. All networks were trained using our modified WTA loss rule. All experiments were performed with a Nvidia Titan X GPU and Tensorflow (Adelphi, MD, USA).

## 6. Results and Discussion

### 6.1. Additional Hypotheses Improve Performance

**Figure 3:**
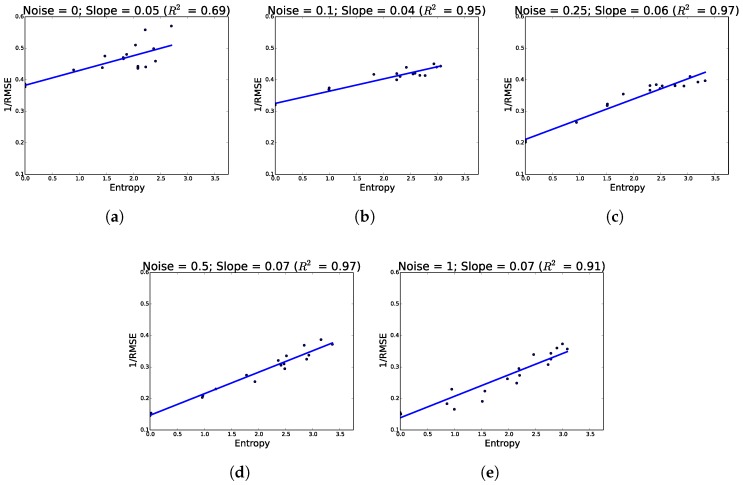
Performance as a function of the entropy of the hypothesis distributions on the test set. For all noise levels, higher entropy corresponds to higher network performance. (**a**) no noise; (**b**) noise = 0.1; (**c**) noise = 0.25; (**d**) noise = 0.5; (**e**) Noise = 1.

As predicted, additional hypothesis pathways improved network performance. Across all conditions, network accuracy increased as a function of hypotheses. Similarly, as we increased noise, networks showed a greater propensity to use their available hypotheses more as evidenced by increasing entropy (see Figure 3 where performance increases as a function of entropy).

### 6.2. Runtime

Table 1 details the comparative runtimes between DM, DSP, and MHDE with various numbers of hypotheses. MHDE runtime scales roughly linearly with number of hypotheses. Overall, the runtime gains of MHDE compared to DM show that providing a strong prior on camera motion allows for far more computationally efficient image predictions and matchings.

### 6.3. Maximum and Active Hypotheses

**Figure 4:**
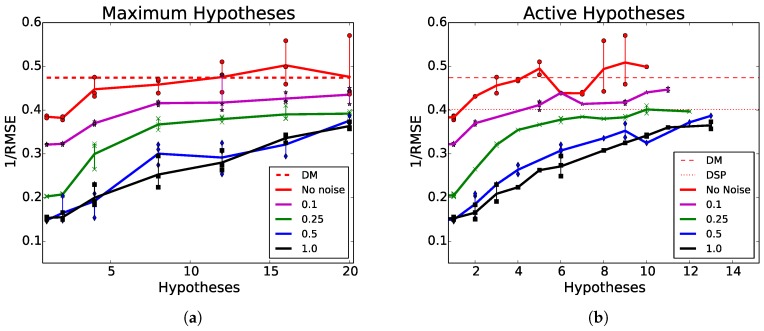
Inverse root mean-squared pixel error (RMSE) (higher is better) for noise conditions 0, 0.1, 0.25, 0.5, and 1 produced by MHDE networks. (**a**) presents results as a function of maximum hypotheses for networks trained with 1, 2, 4, 8, 12, 16, and 20 hypotheses. (**b**) presents the same results as (**a**) but instead plotted as a function of active hypotheses.

**Figure 5:**
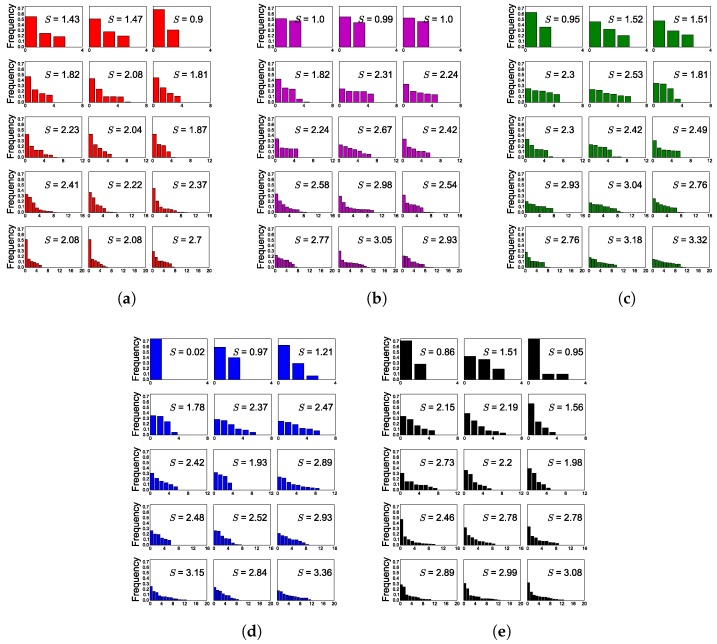
Activation by pathway for the different noise conditions. Only networks with maximum hypotheses of 4, 8, 12, 16, or 20 are shown. (**a**) no noise; (**b**) noise = 0.1; (**c**) noise = 0.25; (**d**) noise = 0.5; (**e**) noise = 1.

Figure 4a shows the performance of MHDE with various maximum hypotheses compared to DM and DSP. A network’s maximum hypotheses is the maximum number of hypothesis generation pathways a given network was allowed to learn. In other words, the number of maximum hypotheses is the total number of hypotheses that a network was initiated with, and, thus, had the option of learning to utilize. Because of our WTA loss rule, this does not mean that the network effectively learned how to use all pathways. For example, in Figure 5d, a network with four maximum hypotheses predominantly trained and used a single pathway.

Figure 4b presents the same results from Figure 4a, but results are plotted as a function of the total active hypotheses instead of maximum hypotheses. Contrastingly, an active hypothesis pathway as one in which that hypothesis pathway produced the lowest error for at least one sample in the test set. Active hypotheses are considered hypothesis pathways that performed better than all other pathways for at least one testing expemplar.

**Figure 6:**
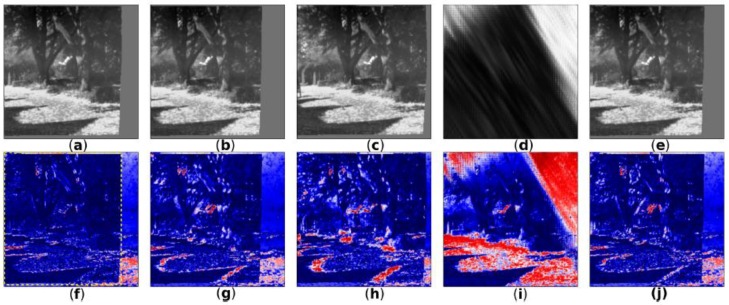
Sample MHDE outputs from different hypothesis pathways. (**a**–**e**): MHDE outputs from five pathways; (**d**) shows the output from an inactive pathway (i.e., a pathway that the network did not optimize); (**f**–**j**): reconstruction error for the hypotheses shown in (**a**–**e**). From (**f**), we can see that the reconstruction shown in (**a**) had the lowest error (yellow-dashed box).

Figure 6 shows five sample reconstructions of a source image by MHDE. For reference, Figure 6D shows the network output of an inactive pathway (or a pathway that the network never learned to properly optimize). While a network may have been trained with a certain number of maximum hypotheses, it may not have learned to use all of them.

Figure 5 shows the activations by pathway for networks trained with 4, 8, 12, 16, and 20 maximum hypotheses. There was a weak relationship between active pathways (pathways that produced the best result for at least one test exemplar) and noise level. The mean number of active pathways were 4.57, 4.95, 5.04, 5.09, and 5.71 for noise levels of 0, 0.1, 0.25, 0.5, and 1, respectively.

There are two main observations from the multi-hypothesis results in Table 1 and Table 2. First, as we increase the number of hypotheses, we see an increase in performance, or, in other words, the more noise, the larger the increase. Second, even in the no-noise case, there still sees an increase in performance. While we had predicted and specifically designed MHDE networks to learn how to sample from an unknown noise distribution, which does seem to be happening, there is also another effect evidenced by the general performance increase across conditions (e.g., the no noise condition).

At first glance, this second effect may have to do with network initialization and early training. However, the variances are actually the lowest in the single hypothesis case and if the networks were becoming less sensitive to initial conditions, this is not what is to be expected. In fact, the exact opposite would be expected.

#### 6.3.1. Network Pruning

There is positive relationship between performance and both maximum hypotheses and active hypotheses. This is true for all noise conditions as well as the no-noise condition. While there is a positive relationship between number of hypotheses and performance for both maximum and active hypotheses, there was less variance when viewing performance as a function of active hypotheses. This indicates that the number of hypotheses actually used by a network is more indicative of performance compared to the number of hypotheses a network is allowed to use. This is interesting because extra hypotheses bring extra computational costs, and if some hypotheses never end up being used, those pathways can be discarded after training and speed up the network. In other words, we can train a network to use some maximum number of hypotheses and once the network has been trained, we can then prune unneeded hypotheses to decrease runtime. For example, we trained a network with 20 maximum hypotheses (shown in Figure 1) that achieved a mean RMSE pixel end-point error of 1.75 on our KITTI test set. This network would run at 75 Hz. However, of the 20 maximum hypotheses, this network only had eight active hypothesis pathways. Because the network structure of MHDE is composable, we can easily prune away the unused pathways to create a network with a total of eight hypotheses pathways, which runs over twice as fast at 158 Hz with identical performance.

## 7. Conclusions

While increased performance in the noise-free conditions was an unintended consequence of the multi-hypothesis formulation, the central contribution of this work is in the handling of noise-contaminated input data. In summary, we have shown the unsupervised learning of correspondence between static grayscale images in a deep sensorimotor fusion network with noisy sensor data. Our network MHDE with 20 hypotheses outperformed DE by 137% in RMSE in our maximum noise condition, by 47% in the noise-free condition, and was 364% slower (357 FPS vs. 76 FPS). Compared to DM, MHDE was 3105% faster with 20 hypotheses (2 FPS vs. 76 FPS) and outperformed DM by 17% in the noise-free condition with 20 hypotheses. In the maximum noise condition, MHDE was outperformed by 23% compared to DM.

We were concerned that MHDE networks might only optimize a single pathway. For example, if one pathway consistently produced the lowest estimate error at the beginning of training, then perhaps only that pathway would be updated and thus the network would not be used to its fullest potential. As seen in Figure 5, this generally was not the case as networks were able to learn to use multiple pathways without intervention outside of the WTA loss rule.

One of the more important aspects of this network is that it does not generate images from scratch and instead works mostly in the space of pixel locations rather than pixel intensities. Given that geometry is consistent across image domains even though image content varies, this network architecture is a promising candidate to leverage transfer learning.

Future work will look at how to include pure sensor measurements (e.g., from an IMU) and how to encourage networks to train and use all available hypothesis pathways. Like its predecessor, MHDE only uses single grayscale images as inputs. Another possible avenue of research is to use multiple images as input, or an LSTM like architecture to give the network additional temporal context.

While we used noise-corrupted motion estimates derived from ground-truth for the MHDE transform input, IMUs are a possible real-world source for this information. However, IMUs only measure accelerations and thus we speculate that using raw IMU measurements as MHDE inputs will result in poor performance during constant velocity maneuvers. Additional work is needed to determine a suitable real-world analog for deriving the motion estimates needed by MHDE.

## Figures and Tables

**Table 1:** Benchmark results for our MHDE networks compared to DeepMatching (DM) [5], and DSP [27] on the KITTI [26] dataset for endpoint error (EPE) with 1–20 hypotheses (H). Mean results are presented from three networks for each condition for the MHDE networks. Min μ is the lowest mean EPE out of the three networks trained for each condition.

		*KITTI (EPE)*			*Runtime*	
Alg.	# H	min μ	μ	σ	ms	FPS
DM [5]	N/A	2.11	2.11	2.69	411	2.4
DSP [27]	N/A	2.49	2.49	4.34	1252	0.8
MHDE	1	2.58	2.6	2.38	**2.8**	**357.1**
MHDE	2	2.59	2.62	2.39	3.3	303
MHDE	4	2.10	2.23	1.88	4.4	227.2
MHDE	8	2.12	2.18	1.72	6.3	158.7
MHDE	12	1.95	2.1	1.69	8.6	116.2
MHDE	16	1.79	**1.99**	1.56	10.8	92.59
MHDE	20	**1.75**	2.1	1.7	13.0	76.92

**Table 2:** MHDE results for heteroscedastic noise conditions on the KITTI [26] dataset for endpoint error (EPE) with 1–20 hypotheses (H).

# H	ϵ	min μ	μ	σ	ϵ	min μ	μ	σ	ϵ	min μ	μ	σ	ϵ	min μ	μ	σ
1	0.1	3.1	3.1	2.9	0.25	4.9	4.9	5.6	0.5	6.6	6.7	9.3	1	6.4	6.5	9.3
2	0.1	3.1	3.1	3.2	0.25	4.8	4.9	5.4	0.5	4.9	6.1	8	1	6	6.4	9.1
4	0.1	2.7	2.7	2.3	0.25	3.1	3.3	3.1	0.5	4.3	5.2	6.5	1	4.4	5	6.2
8	0.1	2.4	2.4	1.9	0.25	2.6	2.7	2.4	0.5	3.1	3.3	3.4	1	3.4	4	4.4
12	0.1	2.3	2.4	2	0.25	2.6	2.6	2.1	0.5	3.1	3.4	4.3	1	3.3	3.6	3.6
16	0.1	2.3	2.3	1.8	0.25	2.4	2.6	2.3	0.5	3	3.1	3.2	1	2.9	3	2.6
20	0.1	2.2	2.3	1.8	0.25	2.5	2.6	2	0.5	2.6	2.7	2.3	1	2.7	2.8	2

## References

[1] Nister D. An efficient solution to the five-point relative pose problem. Proceedings of the 2003 IEEE Computer Society Conference on Computer Vision and Pattern Recognition.

[2] Scharstein D., Szeliski R. (2002). A taxonomy and evaluation of dense two-frame stereo correspondence algorithms. Int. J. Comput. Vis..

[3] Shamwell E.J., Nothwang W.D., Perlis D. DeepEfference: Learning to Predict the Sensory Consequences of Action Through Deep Correspondence. Proceedings of the 2017 IEEE International Conference on Development and Learning and Epigenetic Robotics (ICDL).

[4] Shamwell E.J., Nothwang W.D., Perlis D. A Deep Neural Network Approach to Fusing Vision and Heteroscedastic Motion Estimates for Low-SWaP Robotic Applications. Proceedings of the 2017 International Conference on Multisensor Fusion and Integration for Intelligent Systems.

[5] Revaud J., Weinzaepfel P., Harchaoui Z., Schmid C., Revaud J., Weinzaepfel P., Harchaoui Z., Hi C.S.D., Schmid C. (2016). DeepMatching: Hierarchical Deformable Dense Matching. Int. J. Comput. Vis..

[6] Revaud J., Weinzaepfel P., Harchaoui Z., Schmid C., Revaud J., Weinzaepfel P., Harchaoui Z., Edge C.S.E. (2015). EpicFlow: Edge-Preserving Interpolation of Correspondences for Optical Flow. arXiv.

[7] Kitt B., Moosmann F., Stiller C. Moving on to dynamic environments: Visual odometry using feature classification. Proceedings of the IEEE/RSJ 2010 International Conference on Intelligent Robots and Systems.

[8] Brox T., Malik J., Bregler C. Large displacement optical flow. Proceedings of the CVPR 2009 IEEE Conference on Computer Vision and Pattern Recognition.

[9] Maimone M., Cheng Y., Matthies L. (2007). Two years of visual odometry on the Mars Exploration Rovers. J. Field Robot..

[10] Agrawal M., Konolige K. Real-time localization in outdoor environments using stereo vision and inexpensive GPS. Proceedings of the International Conference on Pattern Recognition.

[11] Enkelmann W. (1991). Obstacle detection by evaluation of optical flow fields from image sequences. Image Vis. Comput..

[12] Davison A.J. Real-time Simultaneous Localisation and Mapping with a Single Camera. Proceedings of the IEEE International Conference on Computer Vision.

[13] Lefaix G., Marchand T., Bouthemy P. (2002). Motion-based obstacle detection and tracking for car driving assistance. Object Recognit. Support. User Interact. Serv. Robot..

[14] Memisevic R. (2013). Learning to relate images. IEEE Trans. Pattern Anal. Mach. Intell..

[15] Memisevic R., Hinton G. Unsupervised Learning of Image Transformations. Proceedings of the 2007 IEEE Conference on Computer Vision and Pattern Recognition.

[16] Ranzato M.A., Hinton G.E. (2010). Factored 3-Way Restricted Boltzmann Machines For Modeling Natural Images. Artif. Intell..

[17] Memisevic R., Hinton G.E. (2010). Learning to represent spatial transformations with factored higher-order Boltzmann machines. Neural Comput..

[18] Hinton G.E., Krizhevsky A., Wang S.D. (2011). Transforming auto-encoders. Lecture Notes in Computer Science (Including Subseries Lecture Notes in Artificial Intelligence and Lecture Notes in Bioinformatics).

[19] Kivinen J.J., Williams C.K.I. (2011). Transformation equivariant Boltzmann machines. Lecture Notes in Computer Science (Including Subseries Lecture Notes in Artificial Intelligence and Lecture Notes in Bioinformatics).

[20] Han S., Mao H., Dally W.J. (2015). Deep compression: Compressing deep neural networks with pruning, trained quantization and huffman coding. arXiv.

[21] Wen W., Wu C., Wang Y., Chen Y., Li H. (2016). Learning structured sparsity in deep neural networks. Advances in Neural Information Processing Systems.

[22] Anwar S., Hwang K., Sung W. (2015). Structured pruning of deep convolutional neural networks. arXiv.

[23] Jaderberg M., Simonyan K., Zisserman A., Kavukcuoglu K. (2015). Spatial Transformer Networks. arXiv.

[24] Glorot X., Bordes A., Bengio Y. Deep Sparse Rectifier Neural Networks. Proceedings of the Fourteenth International Conference on Artificial Intelligence and Statistics.

[25] Abadi M., Agarwal A., Barham P., Brevdo E., Chen Z., Citro C., Corrado G.S., Davis A., Dean J., Devin M. (2016). TensorFlow: Large-Scale Machine Learning on Heterogeneous Systems. arXiv.

[26] Geiger A., Lenz P., Stiller C., Urtasun R. (2013). Vision meets robotics: The KITTI dataset. Int. J. Robot. Res..

[27] Kim J., Liu C., Sha F., Grauman K. Deformable spatial pyramid matching for fast dense correspondences. Proceedings of the IEEE Conference on Computer Vision and Pattern Recognition.

[28] Ciliberto C., Fanello S.R., Natale L., Metta G. A heteroscedastic approach to independent motion detection for actuated visual sensors. Proceedings of the 2012 IEEE/RSJ International Conference on Intelligent Robots and Systems.

